# Increased incidence of pregnancy complications in women who later develop scleroderma: a case control study

**DOI:** 10.1186/ar3510

**Published:** 2011-11-04

**Authors:** Linda van Wyk, Jacolien van der Marel, Annemie JM Schuerwegh, Anne A Schouffoer, Alexandre E Voskuyl, Tom WJ Huizinga, Diana W Bianchi, Sicco A Scherjon

**Affiliations:** 1 Department of Obstetrics, Leiden University Medical Centre, Albinusdreef 2, 2333ZA Leiden, PO BOX 9600, 2300 RC Leiden, The Netherlands; 2 Department of Rheumatology, Leiden University Medical Centre, Albinusdreef 2, 2333ZA Leiden, PO BOX 9600, 2300 RC Leiden, The Netherlands; 3 Department of Rheumatology, VU University Medical Centre, De Boelelaan 1117, 1081 HV, Amsterdam, The Netherlands; 4 Department of Pediatrics, Floating Hospital for Children at Tufts Medical Centre, 755 Washington Street, Boston, MA 02111, USA

**Keywords:** Pregnancy, Chimerism, Systemic sclerosis, Hypertension, Pre-eclampsia, Intra-uterine growth restriction

## Abstract

**Introduction:**

Studies have shown that fetal progenitor cells persist in maternal blood or bone marrow for more than 30 years after delivery. Increased trafficking of fetal cells occurs during pregnancy complications, such as hypertension, preeclampsia, miscarriage and intra-uterine growth restriction (IUGR). Women with these pregnancy complications are significantly more often HLA-class II compatible with their spouses. Women who later develop scleroderma also give birth to an HLA-class II child more often. From these prior studies we hypothesized that preeclampsia and other pregnancy complications could be associated with increased levels of fetal cell trafficking, and later be involved in the development of scleroderma.

**Methods:**

This study was a retrospective multi-centre matched case-control study. One-hundred-and-three women with systemic sclerosis (SSc) and 103 women with no history of SSc or other autoimmune disease were given a questionnaire regarding complications during pregnancy, such as hypertension, intra-uterine growth restriction (IUGR) and miscarriage. Conditional logistic regression analysis was used to assess associations.

**Results:**

We found a statistically significantly increased incidence of having had a pregnancy history of hypertension or a fetus with IUGR in women who subsequently developed SSc compared to healthy controls. We found an odds ratio of 2.6 (95% confidence interval (CI): 1.1 to 4.6) for hypertensive complications during pregnancy and an odds ratio of 3.9 (95% CI: 1.2 to 12.3) for intra-uterine growth restriction for women with SSc compared to healthy controls.

**Conclusions:**

This is the first study to show an association between hypertensive complications during pregnancy or IUGR and the development of SSc at a later age. We speculate that the pregnancy abnormalities may have resulted in increased fetomaternal trafficking, which may have played a role in the increased incidence of SSc. Further studies are indicated to examine this putative relationship.

## Introduction

Systemic sclerosis (SSc) is a connective tissue disease of unknown origin that is characterized by cutaneous and visceral fibrosis, production of auto-antibodies, and prominent microvascular changes. The similarities of this autoimmune disease to graft versus host disease, which is an iatrogenic form of chimerism, have suggested a common pathway in the pathogenesis of both diseases [1].

Increased trafficking of fetal cells into maternal circulation occurs with pregnancy complications such as preeclampsia [2-4]. In preeclampsia, deficient invasion of cytotrophoblasts results in insufficient modification of the maternal spiral arteries. This failure leads to placental hypoxia and placental lesions, which possibly result in increased trafficking of fetal cells into the maternal circulation. Prior studies have shown that fetal DNA levels are significantly higher in pregnancies complicated by intrauterine growth restriction (IUGR), pregnancy induced hypertension and hemolysis elevated liver function low platelet (HELLP) syndrome. As a result of spontaneous miscarriage and after induced abortion, fetomaternal cellular trafficking is also increased [2,3,5-9]. The term microchimerism (MC) refers to the presence of a small population of cells in one individual that have been derived from another genetically distinct person. Well known causes of MC are blood transfusion, transplantation and pregnancy [1]. There is evidence that cells pass through and across the placenta during pregnancy, from mother to fetus and vice versa. Studies have shown that fetal progenitor cells persist in maternal blood or bone marrow for more than 30 years after delivery [10-12].

The increased incidence of autoimmune disease in women after childbearing years, the long-term persistence of fetal progenitor cells in maternal blood and similarities between certain autoimmune diseases and graft versus host disease, have led to the hypothesis that fetal cell microchimerism is somehow involved in the pathogenesis of some forms of autoimmune disease [13]. Male (presumed fetal) DNA and intact cells have been detected more often in the circulating blood and skin of women with SSc as compared to healthy women [5,14].

Several reports have shown that women with preeclampsia and pregnancy induced hypertension are statistically significantly more often HLA-class II compatible with their spouses as compared to women with uncomplicated pregnancies [15]. Women who later develop SSc also give birth to an HLA-class II compatible child more often than healthy women [14,16].

We, therefore, hypothesized that pregnancy complications (for example, PIH, PE, IUGR, miscarriage) could be associated with increased levels of fetal cell trafficking and could later be involved in the development of scleroderma. In order to assess this relationship, we performed a preliminary study to explore such a hypothesis.

## Materials and methods

This retrospective multi-centre matched case-control study was performed at Leiden University Medical Centre (LUMC) and the VU Medical Centre of Amsterdam (VUMC) in the Netherlands. This study was approved by the Medical-Ethical Board of the LUMC.

### Study subjects

A total of 103 cases were recruited from the rheumatology departments of the LUMC and the VUMC. All cases were women with a diagnosis of SSc after their pregnancies had been completed and who had been pregnant at least once. One hundred and three healthy controls with no history of autoimmune disease were recruited from two different general practitioners' clinics in Venhuizen and Noordwijk, The Netherlands. Cases and controls were matched on age (± 5 years) and number of pregnancies (primiparous vs. multiparous).

### Study method

All women received detailed study information and a questionnaire. Informed consent was obtained from all cases and controls. Baseline characteristics, such as maternal age, weight and height, as well as smoking habits during pregnancy and total number of pregnancies were obtained via the questionnaire. Information on the subject's obstetric history, including the sex of their children, and complications during pregnancy was obtained. Women were asked if they had hypertensive complications during pregnancy, such as pregnancy-induced hypertension (PIH) or preeclampsia (PE) (hypertension and proteinuria). Information on the use of anti-hypertensive medication and hospital admission during pregnancy, fetal IUGR and fetal death (miscarriage) was also obtained through the questionnaire. IUGR was defined as abnormal growth of the fetus *in utero*.

### Statistical analysis

Assuming that the incidence of preeclampsia, PIH and IUGR was 25% in the cases and 5% in the controls [17], the power analysis indicated that 110 women in each category were needed to be included so that a power of 0.80 could be reached (α = 0.05). Based on an expected response rate of 80%, 280 questionnaires were sent out by mail to cases (*n *= 140) and controls (*n *= 140).

Baseline characteristics of the cases and controls were compared using the Student *t*-test for means and Chi-square for proportions using SPSS 16.0 (SPSS Inc. Chicago, USA). A conditional logistical regression analysis was used to calculate odds ratios between the cases and the matched controls.

## Results

The response rates to the questionnaire for the cases and controls were 70% and 74%, respectively. There were no differences in demographic characteristics between cases and controls (Table 1). The mean difference between the age at the first pregnancy and the onset of scleroderma was 27.4.

**Table 1:** Baseline characteristics of the cases and their matched controls

Characteristic		Cases *n *= 103	Controls *n *= 103	Mean Difference (95% CI*) or *P*-value
**Age (mean) years**		57.7	57.2	0.5 (-3.6; 2.7)
**Mean age at first pregnancy**		25.6	26.1	-0.5 (-1.0; 1.9)
Mean age at diagnosis (SSc**)		53.0^#^	NA	NA
**Body Mass Index during first pregnancy**		22.2	22.7	-0.5 (-1.4; 0.3)
**Sex of children**	**Male**	30% (31)	19.4% (20)	0.1
	**Female**	16.5% (17)	22.3% (23)	0.4
	**Both**	47.5% (49)	57.3% (59)	0.2
**Number of pregnancies**	**Mean**	2.46	2.41	0.1 (-0.3; 0.2)
	**Primipara**	13.6% (14)	13.6% (14)	1.0
	**Multipara**	86.4% (89)	86.4% (89)	1.0
**Smoking during pregnancy (% yes)**		18% (18.5)	22% (23)	0.5

*CI, confidence interval; **SSc, systemic sclerosis; ^#^*n *= 61 (59.2%), data not complete for all cases

There was a statistically significant association between both hypertensive complications and fetal growth restriction with the development of SSc in later life. Non-statistically significant trends were found in women who experienced miscarriage and those who used anti-hypertensive medication during pregnancy and later developed SSc (Table 2).

**Table 2:** Comparison of obstetric information of the cases and their matched controls

Characteristic	Cases (%)	Controls (%)	*P*-value	Odds Ratio (95% CI*)
**Hypertensive complications during pregnancy**	26.5%	13.7%	0.03	2.6 (1.1 to 4.6)
**Medication use for high blood pressure**	8.8%	3.9%	0.16	2.4 (0.7 to 7.9)
IUGR**	13.7%	3.9%	0.02	3.9 (1.2 to 12.3)
**Miscarriage**	32.7%	21.6%	0.08	1.8 (0.9 to 3.3)

*CI, confidence interval; **IUGR, intra uterine growth restriction.

## Discussion

This preliminary study showed a significant difference in the occurrence of hypertensive complications during pregnancy in women who later developed SSc compared to healthy parous women. There was also a significantly increased incidence of fetal growth restriction in the cases. This is the first study to demonstrate an association between a history of hypertensive complications during pregnancy or IUGR and the development of SSc after childbearing age. The same trend, although not statistically significant, was found for the reported use of anti-hypertensive medication during pregnancy or a history of miscarriage.

The association between hypertensive complications during pregnancy and/or IUGR and subsequent SSc at a later age could possibly be explained by a number of factors. Pregnancy complications, such as hypertension and IUGR, are associated with increased fetal-maternal trafficking [2-4]. Women with SSc, and women with hypertensive complications during pregnancy, are more often HLA-class II compatible with their spouses than controls [18]. Greater tissue antigen compatibility between the pregnant woman and her fetus could also help to explain why fetal cells can persist in the maternal system and can be detected in the mothers' blood and organs for decades post-partum [5,10-12,14]. The similarities between SSc and graft-versus host disease suggests that the pathogenesis of the two diseases might have some similarities. Possibly, pregnancy complications and HLA-class II compatibility lead to increased fetomaternal cell trafficking, which at a later age might be involved in the pathogenesis of SSc. An alternate explanation could be that women who are later destined to develop SSC could already have a sub-clinical form of the disease during pregnancy affecting placental development, which then results in pregnancy complications, such as IUGR and hypertension.

In this study, we obtained information about the subjects' obstetric history by using retrospective questionnaires. Our conclusions are based on the information provided by the research subjects, which is inherently subjective. A more objective approach would have been to review all of the medical records concerning the obstetrical histories for the cases and controls, but this was not possible due to loss of patient files related to the relatively long interval between pregnancy and the development of SSc. Another bias that could affect the results is that in the control group, women with a history of complications during pregnancy could have been more likely to respond to the questionnaire than women with no history of pregnancy complications. However, this potential bias could only result in an underestimation of the association between pregnancy complications and SSc. In addition, patients with a history of scleroderma may recall their medical histories in more detail than controls, which could have led to an overestimation of the studied pregnancy complications. On the other hand, on average, more than two decades passed between the first pregnancy and the age of onset of scleroderma; therefore, this bias is most likely minimal.

## Conclusions

In summary, the results of this preliminary study show that in women who later developed SSc, there was an increased incidence of pregnancy complications, such as hypertension and IUGR. Future studies are indicated to more fully explore this relationship, including its possible association with fetomaternal microchimerism.

## Abbreviations

HELLP: hypertension and hemolysis elevated liver function low platelet; IUGR: Intra-uterine growth restriction; MC: microchimerism; PE: preeclampsia; PIH: pregnancy induced hypertension; SSc: systemic sclerosis.

## Competing interests

The authors declare that they have no competing interests.

## Authors' contributions

SAS conceived the design of the study and drafted the report. AJMS, AAS and AEV recruited participants and provided background knowledge for the data analysis and interpretation. JvM and LvW collected data and drafted the report. TWJH and DB provided background knowledge to the data analysis and interpretation. All authors have reviewed the report. All authors have seen and approved the final version for publication.
